# Supplementary material for: Identification and Functional Analysis of Individual-Specific Subpathways in Lung Adenocarcinoma
Source: Genes (Basel). 2022 Jun 23;13(7):1122. doi: 10.3390/genes13071122 (PMC9315518; doi:10.3390/genes13071122)
Supplement: Supplementary file 1 [file genes-13-01122-s001.zip › genes-1750932-supplementary.pdf]

Table S1 Driver mutation genes in human cancer

Gene Symbol	Gene Symbol	Gene Symbol	Gene Symbol
ABL1	CYLD	KIT	PTEN
ACVR1B	DAXX	KLF4	PTPN11
AKT1	DNMT1	KRAS	RB1
ALK	DNMT3A	MAP2K1	RET
APC	EGFR	MAP3K1	RNF43
AR	EP300	MED12	RUNX1
ARID1A	ERBB2	MEN1	SETD2
ARID1B	EZH2	MET	SETBP1
ARID2	FAM123B	MLH1	SF3B1
ASXL1	FBXW7	MLL2	SMAD2
ATM	FGFR2	MLL3	SMAD4
ATRX	FGFR3	MPL	SMARCA4
AXIN1	FLT3	MSH2	SMARCB1
B2M	FOXL2	MSH6	SMO
BAP1	FUBP1	MYD88	SOCS1
BCL2	GATA1	NCOR1	SOX9
BCOR	GATA2	NF1	SPOP
BRAF	GATA3	NF2	SRSF2
BRCA1	GNA11	NFE2L2	STAG2
BRCA2	GNAQ	NOTCH1	STK11
CARD11	GNAS	NOTCH2	TET2
CASP8	H3F3A	NPM1	TNFAIP3
CBL	HIST1H3B	NRAS	TRAF7
CDC73	HNF1A	PAX5	TP53
CDH1	HRAS	PBRM1	TSC1
CDKN2A	IDH1	PDGFRA	TSHR
CEBPA	IDH2	PHF6	U2AF1
CIC	JAK1	PIK3CA	VHL
CREBBP	JAK2	PIK3R1	WT1
CRLF2	JAK3	PPP2R1A	
CSF1R	KDM5C	PRDM1	
CTNNB1	KDM6A	PTCH1	

Table S2 The potential disease genes in all LUAD samples

Gene	Gene	Gene
PRKX	ITGA2	PLCB2
PLCB3	PIK3CG	ITGA3
PIK3R2	ENTPD3	ITGB1
PIK3CA	PRKACA	NME2
PLCB1	GNGT1	ADCY6
PIK3CB	KRAS	ITGA10
PLCB4	UGT1A5	NME4
AKT2	ADCY9	ADCY3
ITGB4	UGT1A7	AKT1
UGT1A3	ADCY5	NME1
UGT1A9	ENTPD8	ADCY4
ITGAV	UGT1A8	UGT1A6
GNAS	UGT1A10	UGT2B28
ITGA6	PIK3R1	UGT1A5
PIK3R5	PLA2G2F	ADCY9
ITGA8	UGT1A1	UGT1A7
ITGA9	ITGB8	ADCY5
GNG4	PIK3CD	ENTPD8
ADCY2	ITGA1	UGT1A8
AKT3	ITGA11	
